# Case Report: Narcolepsy patients masked behind obstructive sleep apnea syndrome (OSAS): report of 2 cases and literature review

**DOI:** 10.3389/fnins.2025.1563912

**Published:** 2025-04-11

**Authors:** Simin Zou, Xiaomei Zhang, Yinping Shen, Zhongxia Shen, Zhong Wang, Benhong Wang

**Affiliations:** ^1^Department of Psychiatry, Huzhou Third Municipal Hospital, The Affiliated Hospital of Huzhou University, Huzhou, Zhejiang, China; ^2^Peking University Sixth Hospital, Peking University Institute of Mental Health, NHC Key Laboratory of Mental Health (Peking University), National Clinical Research Center for Mental Disorders (Peking University Sixth Hospital), Beijing, China

**Keywords:** narcolepsy, obstructive sleep apnea syndrome, excessive daytime sleepiness, cataplexy, modafinil

## Abstract

Obstructive sleep apnea syndrome (OSAS) and narcolepsy are sleep disorders that commonly present with excessive daytime sleepiness (EDS). OSAS is characterized by recurrent upper airway obstruction during sleep, leading to intermittent hypoxia and sleep fragmentation. Narcolepsy is a chronic sleep-wake disorder characterized by EDS, cataplexy, vivid hallucinations, and sleep paralysis. The overlap of symptoms can lead to misdiagnosis and delayed appropriate treatment. We report two male patients who initially presented with symptoms suggestive of OSAS, including loud snoring, witnessed apneas, and significant daytime sleepiness. Despite appropriate OSAS management with continuous positive airway pressure (CPAP), both patients continued to experience EDS and reported episodes of cataplexy, sleep paralysis, and vivid dreams. Polysomnography (PSG) confirmed mild to moderate OSAS, and multiple sleep latency tests (MSLT) revealed mean sleep latencies of less than 5 min with multiple sleep-onset REM periods (SOREMPs). Based on the presence of cataplexy and MSLT findings, narcolepsy type 1 (NT1) was diagnosed in both cases. Treatment with modafinil in conjunction with CPAP therapy led to significant improvement in symptoms and quality of life. These cases highlight the importance of considering narcolepsy in patients with persistent EDS despite adequate OSAS treatment. Coexistence of NT1 and OSAS can obscure the diagnosis of narcolepsy, leading to delays in appropriate management. Comprehensive evaluation, including detailed patient history and sleep studies, is crucial. Combined therapy targeting both conditions may be effective in managing symptoms and improving patient outcomes.

## 1 Introduction

Obstructive sleep apnea syndrome (OSAS) is a prevalent chronic sleep disorder characterized by periodic obstruction of the upper airway during sleep, leading to reduced or halted airflow and causing intermittent nocturnal hypoxia and sleep fragmentation (Rosenzweig et al., 2015; Marin-Oto et al., 2019). OSAS is commonly associated with excessive daytime sleepiness (EDS), fatigue, and cognitive deficits, making it of significant clinical interest. Research indicates that OSAS affects approximately 9% to 38% of adults globally, reaching nearly 1 billion people, and in some countries, prevalence exceeds 50% (McNicholas et al., 2018; Benjafield et al., 2019; Grote, 2019). EDS is a major symptom of OSAS, manifesting in 40.5% to 58% of patients with the disorder (Bjorvatn et al., 2015). Primary treatments for EDS focus on underlying OSAS, such as the use of continuous positive airway pressure (CPAP), especially in moderate-to-severe OSAS cases (Qaseem et al., 2013). Yet despite its effectiveness, 9% to 65% of patients treated with CPAP continue to experience EDS (Weaver et al., 2007; Koutsourelakis et al., 2009; Pepin et al., 2009; Gasa et al., 2013), and about one-third struggle to maintain treatment adherence (Perez-Carbonell et al., 2022).

Narcolepsy, a sleep-wake disorder lacking specific treatment, the global prevalence is 25–50 per 100 000 people with typical onset during adolescence, although a second peak can occur at ages 30–39 years (Humphreys et al., 2024). It is characterized by EDS, sleep-related hallucinations, and cataplexy, which is a sudden loss of muscle tone triggered by emotions, additional symptoms include sleep paralysis and nocturnal disruptions such as periodic limb movements of sleep (PLMS), restless legs syndrome (RLS), and rapid eye movement sleep behavior disorder (RBD) (Overeem et al., 2001; Dauvilliers et al., 2007; Humphreys et al., 2024; Thorpy et al., 2024). These symptoms are associated with a deficiency of hypocretin-1 neurotransmitters in the cerebrospinal fluid, crucial for regulating wakefulness. According to the International Classification of Sleep Disorders (ICSD-3), narcolepsy is categorized into type 1 (NT1) and type 2 (NT2), with or without cataplexy, respectively. Cataplexy varies in presentation but is often triggered by strong emotions like laughter, anger, or shock, leading to temporary muscle weakness while maintaining consciousness. Despite these varied symptoms, EDS remains the primary and most debilitating symptom (Dauvilliers et al., 2007; Bassetti et al., 2019; Thorpy et al., 2024; Vringer et al., 2024).

Narcolepsy and OSAS are two disorders related to EDS, both contributing to decreased productivity, impaired cognitive function, and reduced quality of life (Lal et al., 2021; Tadrous et al., 2021; Perez-Carbonell et al., 2022). However, these disorders exhibit distinct characteristics. For patients with NT1, EDS is marked by frequent bouts of napping or falling asleep throughout the day. These episodes can be accompanied by muscle weakness, known as cataplexy, which may be triggered by emotions. This often leads to short, refreshing naps associated with vivid dreaming, followed by a return to wakefulness. However, the feeling of sleepiness typically returns within two to three hours (Hoy, 2023). And the severity of OSAS is classified as mild, moderate or severe, different extent of OSAS leads to various impacts on patients with EDS (Wang et al., 2021).

Narcolepsy and OSAS may coexist in the same patient, though the frequency and clinical significance of this overlap are not well understood, this coexistence can delay the diagnosis of narcolepsy and hinder effective treatment (Sansa et al., 2010). Nasal CPAP therapy is the primary treatment for OSAS, involving single- or bi-level positive airway pressure ventilation. Additionally, weight loss through non-surgical and surgical methods is recommended for overweight or obese patients. While these therapies are highly beneficial for treating OSAS, many patients struggle with adherence (Weaver et al., 2007; Patil et al., 2019). Moreover, compliance and tolerance issues can undermine CPAP effectiveness. Consequently, even among those who adhere to CPAP and medication regimens, the persistence of EDS symptoms in some patients indicates that additional symptomatic treatment may be necessary for managing residual EDS (Weaver et al., 2007; Pepin et al., 2009; Gasa et al., 2013).

Fatigue and EDS are the primary complaints among patients with OSAS and narcolepsy (Bailes et al., 2011). Narcolepsy is currently incurable, necessitating lifelong treatment, particularly for NT1 patients (Barateau et al., 2016). Treatment guidelines have been established in Europe and the United States, recommending first-line therapeutic agents for EDS. However, in many Asian countries, central nervous system stimulants like methylphenidate and modafinil are the most accessible options (Chin et al., 2024). Modafinil has been shown to alleviate daytime fatigue and EDS in patients with narcolepsy and OSAS, based on comprehensive research data. While the precise mechanism of action is unclear, it is hypothesized that modafinil enhances arousal-promoting neuronal activity by increasing extracellular dopamine through blockade of dopamine transporters (Chapman et al., 2016; Mohammadi et al., 2021; Hersey and Tanda, 2024; Thorpy et al., 2024). The use of CNS stimulants like modafinil in OSAS patients can obscure the diagnosis of narcolepsy, potentially leading to OSAS being diagnosed while narcolepsy is overlooked. Despite the symptom overlap, narcolepsy and OSAS significantly differ in their etiology, epidemiology, and treatment strategies. Patients with coexisting narcolepsy and OSAS thus require further therapeutic and diagnostic expertise.

**FIGURE 1:**
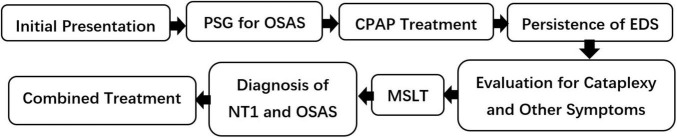
Diagnostic flowchart for suspected coexisting NT1 and OSAS. PSG, polysomnography; CPAP, continuous positive airway pressure; EDS, excessive daytime sleepiness; MSLT, multiple sleep latency test; NT1, narcolepsy type 1; OSAS, obstructive sleep apnea syndrome.

This study aims to highlight the diagnostic challenges of coexisting narcolepsy and OSAS and to explore how frequently narcolepsy is overlooked in OSAS patients, emphasizing the need for improved diagnostic strategies. To assist clinicians in optimizing the diagnostic pathway for suspected comorbid NT1 and OSAS, we have developed a clinical decision-making flowchart that systematically addresses diagnostic challenges in such complex presentations (Figure 1).

## 2 Case description

### 2.1 Case 1

A 30-year-old married man was referred after his wife observed that he regularly exhibited loud snoring, apneic episodes followed by deep inhalation, and occasional shortness of breath during sleep over the past month. The patient himself reported difficulty initiating sleep, poor sleep quality despite sleeping 10–12 h nightly, vivid dreams, episodes of sleep paralysis with auditory hallucinations, and persistent daytime somnolence. On awakening, he often experienced dry mouth, headaches, and episodic hypertension (peaks up to 160/120 mmHg). His body mass index (BMI) was 30.11 kg/m^2^.

Initial evaluations—including blood tests, renal/thyroid/adrenal function tests, video electroencephalogram (EEG), abdominal and carotid ultrasounds, chest computed tomography (CT), and cranial magnetic resonance imaging (MRI)—were unremarkable (with mildly abnormal liver enzymes likely due to poor diet). Psychometric scores were Hamilton Depression Scale (HAMD-17): 7, Hamilton Anxiety Scale (HAMA): 13, Mini-Mental State Examination (MMSE): 27, Pittsburgh Sleep Quality Index (PSQI): 11, and Epworth Sleepiness Scale (ESS): 18. Polysomnography (PSG) confirmed OSAS [total sleep time (TST): 474 min; apnea-hypopnea index (AHI): 11.8; sleep efficiency: 93.3%; minimum oxygen saturation: 85%].

After initiating auto-adjusting CPAP therapy, snoring and apneic events improved (AHI < 5), yet excessive daytime sleepiness (ESS: 14) and episodes of muscle weakness persisted. A subsequent PSG with CPAP and MSLT (average sleep latency: 4.4 min; 3 SOREMPs) together with a 5-year history of involuntary daytime sleep episodes and recent cataplexy (triggered by emotions) led to a diagnosis of narcolepsy type 1 (NT1) combined with OSAS. Treatment with nocturnal CPAP and modafinil 100 mg/day resulted in marked improvement (PSQI: 4; ESS: 3; MMSE: 30) maintained through an 8-week follow-up (Table 1).

**TABLE 1:** Diagnostic PSG, MSLT, and psychometric assessment results for two cases with coexisting NT1 and OSAS.

Parameter	Case 1	Case 2
PSG TST (min)	473.8	463.0
PSG AHI	11.8	15.2
PSG sleep efficiency (%)	93.3	86.9
PSG min O_2_ sat (%)	85	89
MSLT mean sleep latency (min)	4.4	2.6
MSLT SOREMPs	3	5
HAMD-17	7	9
HAMA	13	8
MMSE	27	28
PSQI	11	11
ESS	18	20

PSG, polysomnography; TST, total sleep time; AHI, apnea-hypopnea index; MSLT, multiple sleep latency test; SOREMPs, sleep-onset rapid eye movement periods; HAMD-17, Hamilton Depression Scale; HAMA, Hamilton Anxiety Scale; MMSE, Mini-Mental State Examination; PSQI, Pittsburgh Sleep Quality Index; ESS, Epworth Sleepiness Scale; NT1, narcolepsy type 1; OSAS, obstructive sleep apnea syndrome.

### 2.2 Case 2

A 22-year-old man presented with a two-year history of worsening sleep difficulties, daytime sleepiness, and cognitive issues. He reported light, easily disrupted sleep with loud snoring, and, over the past eight years, persistent daytime fatigue that impacted his concentration and work performance. His symptoms also included vivid dreams, sleep paralysis, and brief episodes of muscle weakness (e.g., sudden finger weakness while using a computer). His BMI had increased from approximately 27.7 to 34.3 kg/m^2^.

Physical, neurological, laboratory, and imaging examinations were normal. Psychometric assessments yielded HAMD-17: 9, HAMA: 8, MMSE: 28, PSQI: 11, and ESS: 20. Initial PSG revealed OSAS (TST: 463 min; AHI: 15.2; sleep efficiency: 86.9%; minimum oxygen saturation: 89%). Following 5 days of CPAP treatment, his OSAS parameters improved (AHI < 5), but excessive daytime sleepiness (ESS: 17), sleep paralysis, and muscle weakness persisted. A repeat PSG with CPAP and an MSLT (average sleep latency: 2.6 min; 5 SOREMPs) confirmed the additional diagnosis of NT1 (Table 1).

Combined CPAP therapy and modafinil 100 mg/day produced significant symptom improvement, with follow-up scores of PSQI: 3, ESS: 4, and MMSE: 30 maintained over 8 weeks.

## 3 Discussion

Narcolepsy is diagnosed through the persistence of EDS and a mean sleep latency of less than 8 min on a MSLT following the exclusion of associated symptoms. Additionally, experiencing two or more SOREMPs is crucial for diagnosis, and NT1 is also characterized by cataplexy (Golden and Lipford, 2018). Studies have shown that patients with narcolepsy are at increased risk for a variety of comorbidities, including obesity, diabetes, depression, thyroid disease, hypertension and eating disorders (Cohen et al., 2018; Baldini et al., 2024). However, OSAS remains the most prevalent comorbidity, both at diagnosis and during follow-up (Sansa et al., 2010; Chang et al., 2023). There is commonly an average delay of 6–10 years before an appropriate diagnosis of narcolepsy, and the symptoms may become less characteristic as patients age (Sullivan, 2010; Frauscher et al., 2013). On average, the diagnostic delay for patients with NT1 extends up to 15 years (Thorpy and Krieger, 2014). In cases of suspected or typical narcolepsy, cataplexy is seldom mentioned, making it likely to be overlooked by clinicians. Studies have reported that the prevalence of OSAS in NT1 patients ranges from 24.8% to 51.4% (Miano et al., 2024). While some suggest that the prevalence of OSAS increases among adults diagnosed with NT1, the intricate mechanisms linking narcolepsy and OSAS, as well as the impact of OSAS on daytime sleepiness, remain areas of ongoing research and have yet to be fully elucidated (Romigi et al., 2021). Moreover, few studies have investigated the comorbidity between pediatric narcolepsy and OSAS; research on the association between narcolepsy and OSAS in children is lacking and yields inconsistent results, complicating diagnosis and treatment (Filardi et al., 2020; Geng and Chen, 2024). The presence of OSAS in pediatric narcolepsy patients may further contribute to the underdiagnosis of narcolepsy. Prolonged diagnostic delays and poor disease management may compound psychosocial challenges in NT1 patients, including reduced educational attainment and employment rates, which are linked to diminished quality of life (Varallo et al., 2024). Therefore, early identification and timely intervention are crucial, as evidenced by cases where symptoms of narcolepsy are obscured by an OSAS diagnosis.

Narcolepsy and OSAS can often be confused because both disorders are associated with EDS as well as an increased body mass index. The MSLT in patients with OSAS may occasionally show two or three SOREMPs, with nocturnal sleep disturbances present in both conditions (Sansa et al., 2010; Golden and Lipford, 2018; Patel, 2019). The mechanisms underlying EDS in CPAP treated patients remain unclear (Lal et al., 2021). As a result, effective wake-promoting agents (WPAs) have emerged as potential adjunctive treatments to improve residual EDS following OSA treatment (Lal et al., 2021; Wang et al., 2024). Regarding narcolepsy, an array of pharmacological options is available, including WPAs, stimulants, and sodium oxybate. However, many patients treated with polypharmacy commonly do not report a complete resolution of EDS symptoms (Thorpy and Dauvilliers, 2015; Thorpy, 2020). In patients with narcolepsy, approximately 30% have obesity, with weight gain often occurring shortly before the onset of symptoms. The incidence of OSAS in this population is about 25%, and nearly half of these patients experience moderate to severe OSAS (Kotagal et al., 2004; Sansa et al., 2010; Frauscher et al., 2013; Meyer and Wittert, 2024), which may explain the low cure rate for narcolepsy. Residual EDS may require other symptomatic treatments, including behavioral strategies such as weight loss, physical activity, avoidance of alcohol, and minimizing sedative use (Wang et al., 2024). These findings indicate the similarities and potential for confusion in the diagnostic and therapeutic complexities of narcolepsy and OSAS.

Modafinil is commonly used as a treatment in Asia and has been approved in the United States to enhance wakefulness in patients experiencing EDS associated with narcolepsy, OSAS, or shift work disorder. In Europe, it is approved for the treatment of EDS in adults with narcolepsy, with or without cataplexy (Arnulf et al., 2023; Chin et al., 2024). Modafinil partially functions by inhibiting the dopamine (DA) and norepinephrine (NE) transporters in the prefrontal cortex, leading to increased levels of DA and NE in the brain. It can act as a cognitive enhancer for healthy adults (Arnulf et al., 2023; Beaudin et al., 2024), and may potentially improve cognitive impairments related to narcolepsy or OSAS in this case. While modafinil carries potential risks of addiction and side effects such as headaches and nausea (Hersey and Tanda, 2024). Though not reported in our case, it is worth noting that some patients have historically found it challenging to adhere to treatment. This underscores the need for a safe and balanced therapeutic strategy. Combining modafinil with CPAP therapy might help reduce the dosage and frequency of side effects associated with its use.

While CPAP therapy is effective in reducing patients’ subjective EDS during the day, the duration of therapy might be insufficient to address underlying structural brain alterations and normalize intracortical inhibition (Castronovo et al., 2014). Limited transcranial magnetic stimulation (TMS) studies in patients with OSAS and narcolepsy have identified abnormalities in cortical excitability (Das et al., 2013). Abnormalities in cortical excitability are thought to underlie the pathophysiology of the various neurocognitive manifestations of OSAS (Das et al., 2013; Lanza et al., 2015; Wang et al., 2023); a similar pattern has been noted in patients with narcolepsy, who exhibit hyperexcitability of excitatory circuits in the motor cortex (increased resting motor threshold and active motor threshold) and increased excitability of inhibitory circuits (Oliviero et al., 2005; Nardone et al., 2011; Huang et al., 2019). Most of the evidence focuses on the prefrontal cortex, especially the left dorsolateral prefrontal cortex (lDLPFC), due to its involvement in emotional regulation, executive functions, and various neural processes, including sleep (Herrero Babiloni et al., 2018; Lanza et al., 2023). The overlap in stimulated brain regions implies that patients with narcolepsy and OSAS may share the same neurobiological disease basis. The use of TMS to assist in the diagnosis and treatment of patients with concurrent narcolepsy and OSAS represents a promising strategy warranting further investigation.

It has been suggested that implementing CPAP therapy during polysomnography (PSG) and the MSLT may enhance the validity of test results (Chervin and Aldrich, 2000). However, the utilization of noninvasive ventilators could potentially alter sleep latency, thus influencing MSLT outcomes, warranting further investigation into this methodology. Misdiagnosis of narcolepsy in cases where EDS is primarily due to untreated or undertreated OSAS is a significant concern, as it can lead to PSG findings similar to those in narcolepsy (Chang et al., 2023). OSAS frequently co-occurs in patients with narcolepsy and is a more prevalent cause of EDS. Consequently, in instances of EDS, it is essential to confirm the efficacy of OSAS treatment before excluding a narcolepsy diagnosis.

Narcolepsy’s debilitating effects significantly disrupt social and occupational functioning in nearly half of the affected individuals (David et al., 2012; Bassetti et al., 2021). Furthermore, patients with sleep-wake disorders, which are notably characterized by EDS, face an elevated risk of sleep-related accidents (Philip et al., 2010; Tzeng et al., 2019; Chien et al., 2022), as described in our case that there is a risk of sleep-related accidents. This individual risk underscores the clinical imperative to manage narcolepsy symptoms, particularly EDS. Non-pharmacologic interventions, such as regular naps and improved sleep hygiene, can partially alleviate EDS symptoms (Golden and Lipford, 2018). However, pharmacologic treatments, when combined with CPAP therapy, may form the cornerstone for effectively treating narcolepsy co-occurring with OSAS.

In patients with OSAS, it is essential to actively search for symptoms of cataplexy to rule out narcolepsy. CPAP therapy does not typically alleviate EDS in patients who have both narcolepsy and OSAS (Sansa et al., 2010). Therefore, these patients may require additional targeted treatment to address the residual EDS following primary interventions. Care must be taken with the use of central nervous system (CNS) stimulants, such as modafinil, for improving EDS in OSAS patients, as it may obscure the diagnosis of narcolepsy, modafinil’s ability to enhance wakefulness can inadvertently conceal the underlying symptoms of narcolepsy. Although CNS stimulants and CPAP can be effective in ameliorating symptoms of OSAS and its resultant EDS for substantial periods, overlooking narcolepsy can negatively impact the management and prognosis of the condition. Current focus tends to be on EDS in the student population, often neglecting adult cases. For instance, two adult patients recently exhibited significant life-altering effects from EDS, highlighting the need for further advancements and attention in sleep medicine.

## 4 Conclusion

These cases highlight the importance of considering narcolepsy in patients with persistent EDS despite adequate OSAS treatment. Various factors underscore the importance of remaining vigilant regarding the co-occurrence of OSAS and narcolepsy. When treatment of OSAS yields no or minimal improvement in EDS, healthcare professionals should evaluate the likelihood of an additional sleep disorder, such as narcolepsy or other central disorders of hypersomnolence. If, following treatment, patients continue to experience EDS, despite quantitative assessments like PSG and the MSLT, it may be appropriate to consider the presence of narcolepsy, and caution in the premature use of CNS.

For patients with both narcolepsy and OSAS, although OSAS is mildly (as presented in this case, 5 < AHI < 15), a timely CPAP+modafinil combination approach may be a treatment strategy that is relatively fast-acting and avoids the development of residual EDS. Future research should focus on elucidating the mechanisms underlying the coexistence of narcolepsy and OSAS and developing advanced diagnostic tools to differentiate these disorders more effectively. Clinicians are encouraged to routinely evaluate for narcolepsy in appropriate cases, particularly when EDS persists after OSAS treatment.

### 4.1 Limitations

Limitations of this study include the absence of a controlled trial and the inability to measure cerebrospinal fluid (CSF) hypocretin-1 levels due to patient refusal, a significant constraint given its diagnostic value for NT1. Serum HLA typing, while associated with NT1, lacks specificity for diagnosis and was not utilized in this study. Reliance on subjective patient reports introduces potential bias, and the small sample size limits generalizability. Uncertainty persists regarding whether narcolepsy or OSAS symptoms are primary or secondary, and the underlying neuropathological mechanisms remain unclear. Additionally, incorporating physical therapies such as TMS represents a potential non-invasive diagnostic method and treatment strategy. Future research should incorporate CSF testing where feasible and larger cohorts to validate findings and reduce bias.

## Data Availability

The original contributions presented in this study are included in this article/supplementary material, further inquiries can be directed to the corresponding authors.
